# Inert Tubercle Bacilli—Other More Dangerous Organisms in the Sputum and Lungs

**Published:** 1892-06

**Authors:** F. J. Thornbury

**Affiliations:** 610 Main Street; Buffalo, N. Y.


### INERT TUBERCLE BACILLI—OTHER MORE DANGEROUS ORGANISMS IN THE SPUTUM AND LUNGS.

By F. J. THORNBURY, M. D., Buffalo, N. Y.

KITASATO was recently assigned, by Prof. Koch, the work of cultivating tubercle bacilli from the sputum, and contents of closed tubercular cavities.

As well known, many, and the French in particular, have rather doubted the possibility of cultivating tubercle without first passing it through the body of animals. The cultivation, however, can be accomplished without this re-inoculation, although it is attended with difficulty, by reason of the slow growth of the tubercle bacillus and the luxurious development of other germs from the mouth. Koch's suggestion of using the early morning expectorate was employed, and from the receptacle containing some of this sputum, the numular, cheesy particles coming from deep in the lungs were fished out and deposited in Petrie's shallow plates containing water. These isolated particles are washed carefully in ten successive Petrie's glasses. In this manner all the organisms which have become adherent in passing through the mouth, are removed. The sputum is then teased in the Petrie's glasses under sterilized water, and a particle taken from the middle, a nodule, is stained and examined under the microscope to determine if tubercle bacilli are actually present. Very often they are, and then it is only necessary to transfer a particle of the sputum on glycerine agar or blood serum to obtain pure cultures of the bacillus. The cultures from the sputum differentiate themselves in appearance at the beginning of their growth from the cultures of bacilli obtained from organs. In each instance, the colonies first commence to form in about two weeks, but they present an entirely different appearance. The sputum colonies occur as clear, elevated, translucent spots. In addition, they are moist, glistening and smooth,—much like the colonies of white yeast; while the colonies representing organ cultures appear from the first dry, insipid and nodular. Gradually this difference subsides so that after four weeks the contrast between the two growths is no longer present,—a consequence naturally to be expected, as the sputum is nothing else than the fluid expectorated from tubercular cavities. Kitasato has also been successful in obtaining from closed lung cavities, post-mortem, many other bacteria found associated with tubercle, and which seem to play an exceedingly important roll in the course of the disease.

Cultivation experiments further demonstrate the interesting fact that many of the tubercle bacilli found in the sputum and lungs are inert and quite harmless, although microscopically no difference from live bacilli is recognizable. The dead bacilli color just as quickly and intensely and cannot be discriminated morphologically. But the fact of their being inert can be demonstrated in the following manner: A quantity of the sputum, or pus from a tubercular cavity, is spread on the surface of agar and the tube capped. The sputum remains visable for weeks and the tube shows no tubercular growth, while others of the same series present colonies characteristic of tubercle.

A specimen of the sputum from the sterile agar-tube is now examined microscopically. Myriads of fine appearing, readily stainable tubercle bacilli will be seen. With the sputum containing these bacilli which have remained inert in the agar-tube kept in the incubator for a long time, guinea-pigs inoculated died in two weeks. *But not one of them showed a trace of tuberculosis.* This demonstrates *conclusively* that the vast majority of tubercle bacilli occurring in the sputum and lungs are *inert*, and that this loss of vitality is not recognizable microscopically as yet. With reference to the other bacteria associated with tubercle in the sputum, the presence of certain forms is constant and their pathological significance *must be inferred.* In fact, when the case comes to autopsy, the organisms which were present in the sputum are found disseminated in pure cultures *through* the lungs, and indeed, in one case, (in this instance a bacillus,) the previously seen organism was also found in pure cultures *in every visceral organ of the body.* Many varieties of these bacteria may be cultivated, which during life are constantly associated with the tubercle bacillus, and post-mortem, are found in *pure cultures* in the lungs. There are three forms discovered by Kitasato, two different streptococci and three micrococci. These organisms fluctuate in quantity, like the tubercle bacilli themselves, as shown by continuous microscopic *examination.* I have, myself, recently observed in examination of tubercular sputum, masses of streptococci and micrococci vastly in predominance of the isolated and scattered tubercle bacilli. These strange organisms show simply the *counter-stain* of methyl-blue, while the tubercle bacilli, themselves, *alive or inert,* take the fuchsine red, and have the usual characteristic appearance.

610 Main Street.

